# Roles considered important for hospitalist and non-hospitalist generalist practice in Japan: a survey study

**DOI:** 10.1186/s12875-023-02090-w

**Published:** 2023-07-07

**Authors:** Taiju Miyagami, Taro Shimizu, Shunsuke Kosugi, Yohei Kanzawa, Kazuya Nagasaki, Hiroyuki Nagano, Toru Yamada, Kazutoshi Fujibayashi, Gautam A. Deshpande, Flora Kisuule, Susumu Tazuma, Toshio Naito

**Affiliations:** 1 Department of General Medicine, Juntendo University Faculty of Medicine, Tokyo, Japan; 2 Department of Diagnostic and Generalist Medicine, Dokkyo Medical University Hospital, Kitakobayashi 880, Mibu, Shimotsuga, Tochigi 321-0293 Japan; 3 Department of General Internal Medicine, Iizuka Hospital, Iizuka, Fukuoka Japan; 4 Department of General Internal Medicine, Akashi Medical Center, Hyogo, Japan; 5 Department of Internal Medicine, Mito Kyodo General Hospital, University of Tsukuba, Ibaraki, Japan; 6 Department of Healthcare Economics and Quality Management Graduate School of Medicine, Kyoto University, Kyoto, Japan; 7 Department of General Medicine, Graduate School of Medical and Dental Sciences, Tokyo Medical and Dental University, Tokyo, Japan; 8 Division of Hospital Medicine at Johns Hopkins Bayview Medical Center, Baltimore, MD USA; 9 JA Onomichi General Hospital, Hiroshima, Japan

**Keywords:** Hospitalist, General medicine, Questionnaire, Non-hospitalist generalist

## Abstract

**Background:**

An increased focus on quality and patient safety has led to the evolution of hospitalists. The number of hospitalists covering ward and outpatient care is on the rise in Japan. However, it is unclear what roles hospital workers themselves consider important in their practice. Therefore, this study investigated what hospitalists and non-hospitalist generalists in Japan consider important for the practice of their specialty.

**Methods:**

This was an observational study that included Japanese hospitalists (1) currently working in a general medicine (GM) or general internal medicine department and (2) working at a hospital. Using originally developed questionnaire items, we surveyed the items important to hospitalists and non-hospitalist generalists.

**Results:**

There were 971 participants (733 hospitalists, 238 non-hospitalist) in the study. The response rate was 26.1%. Both hospitalists and non-hospitalists ranked evidence-based medicine as the most important for their practice. In addition, hospitalists ranked diagnostic reasoning and inpatient medical management as the second and third most important roles for their practice, while non-hospitalists ranked inpatient medical management and elderly care as second and third.

**Conclusions:**

This is the first study investigating the roles Japanese hospitalists consider important and comparing those to that of non-hospitalist generalists. Many of the items that hospitalists considered important were those that hospitalists in Japan are working on within and outside academic societies. We found that diagnostic medicine and quality and safety are areas that are likely to see further evolution as hospitalists specifically emphasized on them. In the future, we expect to see suggestions and research for further enhancing the items that hospital workers value and emphasise upon.

**Supplementary Information:**

The online version contains supplementary material available at 10.1186/s12875-023-02090-w.

## Background

In the mid-1990s, an increased focus on quality and patient safety led to the evolution of the hospitalist specialty [1, 2]. Since its inception, the number of hospitalists has grown, with current estimates of at least 44,000 non-paediatric hospitalists in the United States of America (USA) [3]. An interesting aspect of the rapid expansion of hospital medicine is the growth of the field beyond USA, including Asian Countries [4–6]. While some of the reasons for the development of hospital medicine internationally are the same as in the United States, there are differences in training, healthcare systems, regulations, and cultural norms, underscoring the practice of hospital medicine in different countries. In Japan, the number of hospitalists is increasing; however, their role and importance is unclear as of 2022. This survey study investigated what hospitalists and non-hospitalist generalists in Japan consider important for the practice of their specialty.

In Japan, post-graduate year (PGY)1 − 2 residents spend the first two years rotating through various specialties such as internal medicine, general medicine (similar to family practice in the United States), paediatrics, obstetrics and gynaecology, and emergency medicine after graduation from medical school [7]. In PGY 3 − 5, residents pick an area of specialisation [8]. In 2018, post-graduate training in Japan was modified to incorporate hospital medicine [8]. There are now two pathways through which Japanese doctors can become hospitalists. PGY 3 − 5 residents specialising in either general internal medicine or general medicine can go on to become hospitalists. Specialists in general internal medicine, general medicine, and hospital medicine are all considered generalists (Appendix 1). Until recently, the definition of general medicine in Japan has been ambiguous, and there has been no clear distinction between the role of a hospitalist and a non-hospitalist generalist [9, 10]. The historical lack of role clarity has influenced the practice of hospitalists in Japan, who, unlike many hospitalists in the U.S., do not work exclusively in hospitals. Instead, Japanese hospitalists take care of patients in multiple locations at hospitals (over 20 beds), outpatient clinics, emergency rooms, and ICUs. Non-hospitalist generalists are often described as family physicians. They often work in clinics with no beds or fewer than 20 beds. They specialise in treating different patients and problems from a broad perspective, not limited to a specific disease or age group. While emphasising family relationships, they must be able to comprehensively address health issues commonly encountered in the community regardless of age or disease, including preventive care, multimorbidity, and psychosocial issues [11, 12].

Most of the hospitalist and non-hospitalist generalists in Japan belong to one (or both) of two societies: The Japan Primary Care Association (JPCA) and the Japanese Society of Hospital General Medicine (JSHGM). In 2022, the JPCA became a professional organisation certifying family physicians, while JSHGM is the entity for certifying hospitalists [13, 14]. Membership in an academic society is a prerequisite for both specialties. As a professional and certification organisation for hospitalists, JSHGM has described the 10 most important roles for the Japanese hospitalist [15]. These include a generalist mindset, leadership, management, comprehensive community care, collaboration with multiple professions, medical interviews, physical examinations, diagnostic Reasoning, and active educational and academic activities [15, 16]. These roles are akin to the 24 “core competencies” for hospitalists in the U.S. defined by the Society of Hospital Medicine (SHM) [17].

Although these priorities have been defined by their professional organisations in Japan and the U.S., it is unclear what roles hospitalists themselves consider important in their practice. It is also unknown whether clinical priorities for hospitalists differ from those of non-hospitalist generalists. Clarity around practice is important in differentiating the professional differences between hospitalists and non-hospitalist generalists and will facilitate the continued training and development of hospitalists in Japan. Therefore, we performed this survey study to investigate what hospitalists and non-hospitalist generalists in Japan consider important for the practice of their specialty.

## Methods

### Setting and participants

This study was an observational survey, based with questionnaires sent to all hospitalists and non-hospitalist generalists listed in the JPCA and JSHGM mailing lists. For this study, hospitalists were defined as general medicine physicians working in hospital wards or in a clinical practice affiliated with a medium to a large hospital. The response period was from January 28 to March 28, 2020. Residents were excluded from the study [17, 18]. We used the Research Electronic Data Capture to store data online [19].

### Survey instrument

Subject matter experts (TM, SK, YK, KN, HN, and GD) met in a series of rapid cycle sessions to generate and revise questions for the survey. This content expertise was based on: (1) approximately 10 years of hospitalist practice in Japan, (2) experience working as a hospitalist in the United States (GD), and (3) involvement in creating competencies for hospitalists in Japan (TS and TN). The content experts first determined the possible roles of Japanese hospitalists based on the 10 items considered important by the JSHGM [15] and the 24 items included in the core competencies for hospital medicine in health care systems for hospitalists in the U.S. as described by SHM [17]. Twenty-six items were ultimately included in the survey. Respondents were asked to select the three items they thought were the most important roles for hospitalist practice, ranking them first to third. Responses were scored by applying 3 points to the first ranked item, 2 points to the second, 1 point to the third, and 0 points for “not applicable.” This scoring method was created based on the Borda count [19, 20]. The scores for each item were then divided by the overall total score and expressed as a percentage. The survey also included demographics questions (age and sex), post-graduate years at the time of the baseline survey, academic society memberships (JPCA and JSHGM), whether they belonged to a general medicine department in a school of medicine, practice setting (university hospital, community-based hospital, clinic), and hospital size. Institutions with 19 or fewer beds were referred to as clinics, and institutions with 20 or more beds were referred to as hospitals, according to the standards of the Ministry of Health, Labour and Welfare [19, 21]. A sub-analysis, comparing the scores, was performed for community-based and university hospitals. For this study, hospitals with 20 − 199 beds, 200 − 399 beds, and > 400 beds were categorised as small, medium-sized, and large hospitals, respectively. Respondents were categorised as residents, attendings, managers, and “other” (Table 1). Doctors who attended a specialty program for three years (PGY3 − 5) were called residents. Staff members were called attendings, managers were called managers, and physicians who did not fit into any preceding category were called others. The survey was conducted in Japanese, and the English version of the questionnaire was published as an appendix (Appendix 2).

**Table 1:** Participant characteristics

Participant characteristics	Hospitalist group Total *N* = 733 N (%)	Non-hospitalist group Total *N* = 238 N (%)	*P* value
Male	610 (83.2)	185 (77.8)	0.010
Age, median (range)	46 (37,55)	45 (38,55)	0.899
Post-graduate year, median (range)	20 (11,30)	19 (12,28)	0.943
Belong to JPCA^a^	553 (75.4)	226 (95.0)	< 0.0001
Belong to JSHGM^b^	468 (63.9)	34 (14.2)	< 0.0001
Belong to JPCA and JSHGM	295 (40.2)	23 (9.7)	< 0.0001
Work Environment			
Community-based hospital	520 (70.9)	N/A	
University hospital	196 (26.7)	N/A	
No. beds of primary hospital^c^			
Small hospital 20–199	192 (26.2)	N/A	
Medium hospital 200–399	201 (27.4)	N/A	
Large hospital ≥ 400	323 (45.1)	N/A	
Position			
Resident of specialty training program	76 (10.3)	11 (4.6)	< 0.0001
Attending	392 (53.5)	46 (19.3)	< 0.0001
Manager	224 (30.6)	152 (63.9)	< 0.0001
Others	41 (5.6)	29 (12.1)	< 0.0001

Values are presented as medians (interquartile range) or number (%)

^a^*JPCA* Japanese primary care association

^b^*JSHGM* Japanese society of hospital general medicine

^c^Some participants “’” were not aware of the number of their primary hospitals

### Data analysis

Results are presented as medians (interquartile range) for continuous variables or prevalence (%) for categorical variables. All calculations were performed using JMP PRO software, version 13.0 (SAS Institute, Cary, NC, USA). Variables were subjected to the chi-square test, and *p* values < 0.05 were considered statistically significant.

## Results

**Fig. 1:**
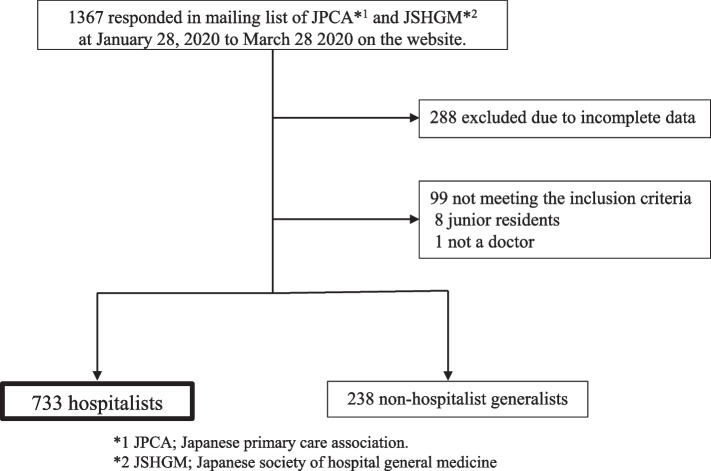
Flow chart of participants in this study. From 1,367 survey respondents, 971 were included in the study. Of these, 733 were in the hospitalist group and 238 were in the non-hospitalist group

The number of physicians with known affiliations on these two mailing lists was 3,466 for JPCA and 1,766 for JSGHM. Several participants were members of both associations but only responded to the questionnaire once. There were 1,367 unique respondents, and the overall response rate was 26.1%. Two hundred eighty-eight incomplete surveys were excluded from the analysis. Nine respondents did not meet the inclusion criteria (eight were residents, and one was not a physician). A total of 733 hospitalists and 238 non-hospitalist generalists were included in the study (Fig. 1).

In the hospitalist group, 85.2% were men, and the median age was 46 years. Further, 41.2% belonged to both JSHGM and JPCA; 72.6% of the respondents worked in community-based hospitals, 45.1% worked in large hospitals, and 53.5% were attendings. There were no significant differences between the hospitalist and non-hospitalist groups regarding age and post-graduate year of practice (Table 1).

**Fig. 2:**
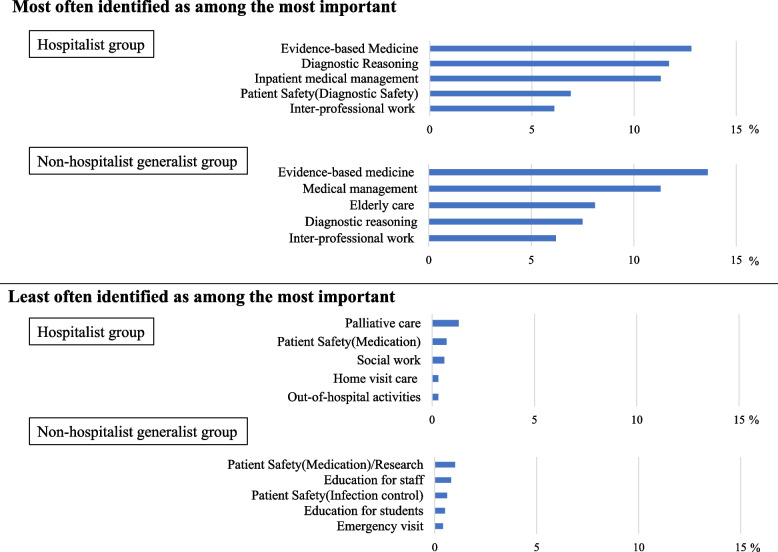
Please choose what is the most important to you as a hospitalist. Respondents were asked to select each of the three items they thought were most important roles for hospitalist practice, ranking them first to third. Responses were scored by applying 3 points to the first ranked item, 2 points to the second, 1 point to the third, and 0 points for “not applicable.” This scoring method was created based on the Borda count. The most and least often identified as among the most important of each group are listed

Both hospitalists and non-hospitalists most frequently ranked evidence-based medicine among the three most important roles. However, hospitalists ranked diagnostics and inpatient medical management as the second and third among the three most important roles, while non-hospitalists ranked inpatient medical management and elderly care as the second and third among the three most important roles. While there were similarities in what the generalists ranked as important, the roles least often identified as among the most important differed in the two groups (Fig. 2) (Appendix 3). Interestingly, in a comparison of community-based hospitals and university hospitals, hospitalists at community-based hospitals placed the highest importance on inpatient medical management (13.2% (6.2% in the university hospital group)), while hospitalists at university hospitals placed the highest importance on diagnostic reasoning (15.2% (10.3% in the community-based hospital group)) (Appendix 4). Regarding gender comparison, males rendered the most importance to evidence-based medicine (13.0% [11.5% in the female group]), while females gave the most importance to inpatient medical management (14.8% [10.7% in the male group]) (Appendix 5). According to position, residents and attending respondents gave the highest importance to evidence-based medicine (resident 17.1%, attending 13.2%), while managers accorded the highest importance to diagnostic reasoning (11.5%) (Appendix 6). In comparison by academic society, those belonging to only JPCA regarded inpatient medical management as the most important (14.1%), while those belonging to only JSHGM regarded evidence-based medicine as the most important (15.5%). Those who belonged to both societies considered diagnostic reasoning the most important (13.0%) (Appendix 7).

## Discussion

This study is the first to investigate what roles Japanese hospitalists consider important for their practice. Japanese and non-hospitalist generalists ranked evidence-based medicine, diagnostic reasoning, and inpatient medical management as the most important roles for their practice. The relative importance of evidence-based medicine, diagnostic reasoning, and inpatient medical management for both hospitalists and non-hospitalist generalists is congruent with clinical practice in Japan. In addition, in a study of Japanese general medicine doctors, clinical care was the most important among the four categories of clinical care, education, research, and management [22]. This suggests that both hospitalists and non-hospitalist generalists may also emphasise on clinical care. A comparison of community-based hospitals and university hospitals among hospitalists showed that community-based hospitals placed more importance on inpatient medical management. In comparison, university hospitals placed more importance on diagnostic reasoning. This may reflect the large number of hospitalists in this study who belonged to community-based hospitals. This may also be influenced by previous reports that university hospitals tend to receive consults for difficult-to-diagnose cases [23]. Moreover, there is a long-standing history of training diagnostic thinking in Japan. This is accomplished through collective intelligence and non-profit conferences on diagnostic reasoning frequently held in Japan [24, 25]. In 2019, JSGHM created a working group on diagnostic excellence to evolve the area of diagnostic medicine in Japan, and this group has been very active to date [26, 27]. Within JSGHM conferences, the group reports the largest number of cases and conducts workshops on diagnostic medicine. Outside of the JSGHM, the group has produced the most research in Japan in this field.

Hospitalists prioritise the quality and safety of medical care over non-hospitalist generalists. Studies have shown that the presence of hospitalists in Japan has shortened hospital stays and reduced costs for common diseases such as pneumonia and heart failure [28–30]. Similarly, a report summarising the role of hospitalists in four Asian countries also emphasises the importance of their role in addressing healthcare quality [5]. The results of these previous studies indicate that medical care quality and safety are core interest for Japanese hospitalists and that their work improves patient care in this area.

Our study also shows that non-hospitalist generalists in Japan place more importance on elderly care compared to that by hospitalists. When the frequency of inpatient medical management and the frequency of elderly care are added together, there may not be a large difference. Still, the care of the elderly is important to hospitalists. Japan is an aging society, with 25% of the population over 65 years old as of 2013, and this figure is expected to reach 40% by 2060 [31]. Elderly people are more likely to have multimorbidity, and 62.8% of those aged 65 years and older present with multiple co-morbidities [32]. Korean hospitalists reportedly contribute to shorter hospital stays for this patient population [33]. In addition, a previous report recommends that hospitalists in Europe, where the proportion of elderly patients is smaller than in Japan, need to pay more attention to diseases related to the elderly [34]. We believe that the care of the elderly is a crucial area of development for hospitalists in the future. In order to distinguish between hospitalists and non-hospitalist generalists, JSHGM believes that the original criteria could be refined to better define the role of the hospitalist [15].

This study has several limitations. First, the response rate was low at 26.1%. However, our survey was sent to all non-unique members of JPCA and JSHGM; therefore, our response rate is underestimated. In addition, a large number of respondents dropped out of the survey, 288, of which 281 were nearly non-responsive, which is a very large number. This may be because the survey was an online survey that could be answered from a cell phone, and therefore many respondents may have been interrupted by other tasks or may have opened the survey once and answered later. Moreover, those who respond to a research-based survey like this may have a high affinity for research among general medical doctors. However, this study did not examine the respondents' backgrounds; thus, further research is needed. Second, we only surveyed Japanese hospitalists and non-hospitalist generalists who are members of JPCA and JSHGM; hence, there is a high possibility of response bias. Further, their perspectives do not necessarily represent those of the broader population of Japanese generalists. Third, the evaluation is based on a ranking system. As a result, the interval between the first, second, and third places in the chosen ranking is not necessarily the same. Fourth, the survey items were slightly skewed, and the fact that some items were detailed, and others were not may have affected the results of this survey. Fifth, participants in this study differed by gender, position, and academic affiliation, and this bias may have affected the results. Sixth, this survey was conducted exactly when the COVID-19 epidemic began to spread in Japan. Perhaps, this timing may have influenced the responses, as the results of the questionnaire also focused on the increasing intensity of public health messages and the increasing clinical evidence on COVID-19; however, this is one of the only viable ways to characterise informants’ perspectives. Despite these limitations, this study is the first to evaluate what Japanese hospitalists consider important roles in their practice. This study could be an important contribution highlighting key areas for the professional development of the hospital medicine profession in Japan and globally.

## Conclusion

Japanese hospitalists practice in multiple settings, and there is some ambiguity around the uniqueness of their role compared to non-hospitalist generalists. While JSHGM has defined some core competencies for their practice, it is not yet known what Japanese hospitalists consider important for their practice. These findings call attention to current gaps in the top three priorities of hospitalists. They may be useful in investigating whether and how hospitalist priorities differ from core competencies and other requirements that determine whether training and practice activities should be renewed. Further research is needed to look at comparisons in practice and attitudinal similarities and differences in what hospitalists around the world consider important roles in their practice. Opportunities for enhancing hospitalist practice in the areas identified as important for their practice would also be a significant next step. This work would be crucial in fostering collaboration in training and practice development to build a strong specialty that leverages the unique characteristics of the international hospitalist community.

## Supplementary Information

**Additional file 1.** 

**Additional file 2: Appendix 2.** Questionnaire raw data English Ver.

**Additional file 3: Appendix 3.** Questionnaire resultwhat you consider important as a hospitalist?

**Additional file 4: Appendix 4.** Compare community-based hospital with university hospital.

**Additional file 5: Appendix 5.** Compare male with female.

**Additional file 6: Appendix 6.** Compare resident of specialty training program, attending with manager.

**Additional file 7: Appendix 7.** Comparison of Academic Affiliations.

## Data Availability

The datasets used and analysed during the current study are available from the corresponding author on reasonable request.

## Abbreviations

JPCA: Japan primary care association
JSHGM: Japanese Society of Hospital General Medicine
SHM: Society of hospital medicine
ICU: Intensive care unit
PGY: Post-graduate year
