# Supplementary material for: Roles considered important for hospitalist and non-hospitalist generalist practice in Japan: a survey study
Source: BMC Prim Care. 2023 Jul 7;24:139. doi: 10.1186/s12875-023-02090-w (PMC10327327; doi:10.1186/s12875-023-02090-w)
Supplement: Supplementary file 1 — Additional file 1. [file 12875_2023_2090_MOESM1_ESM.pptx]

## Slide 1
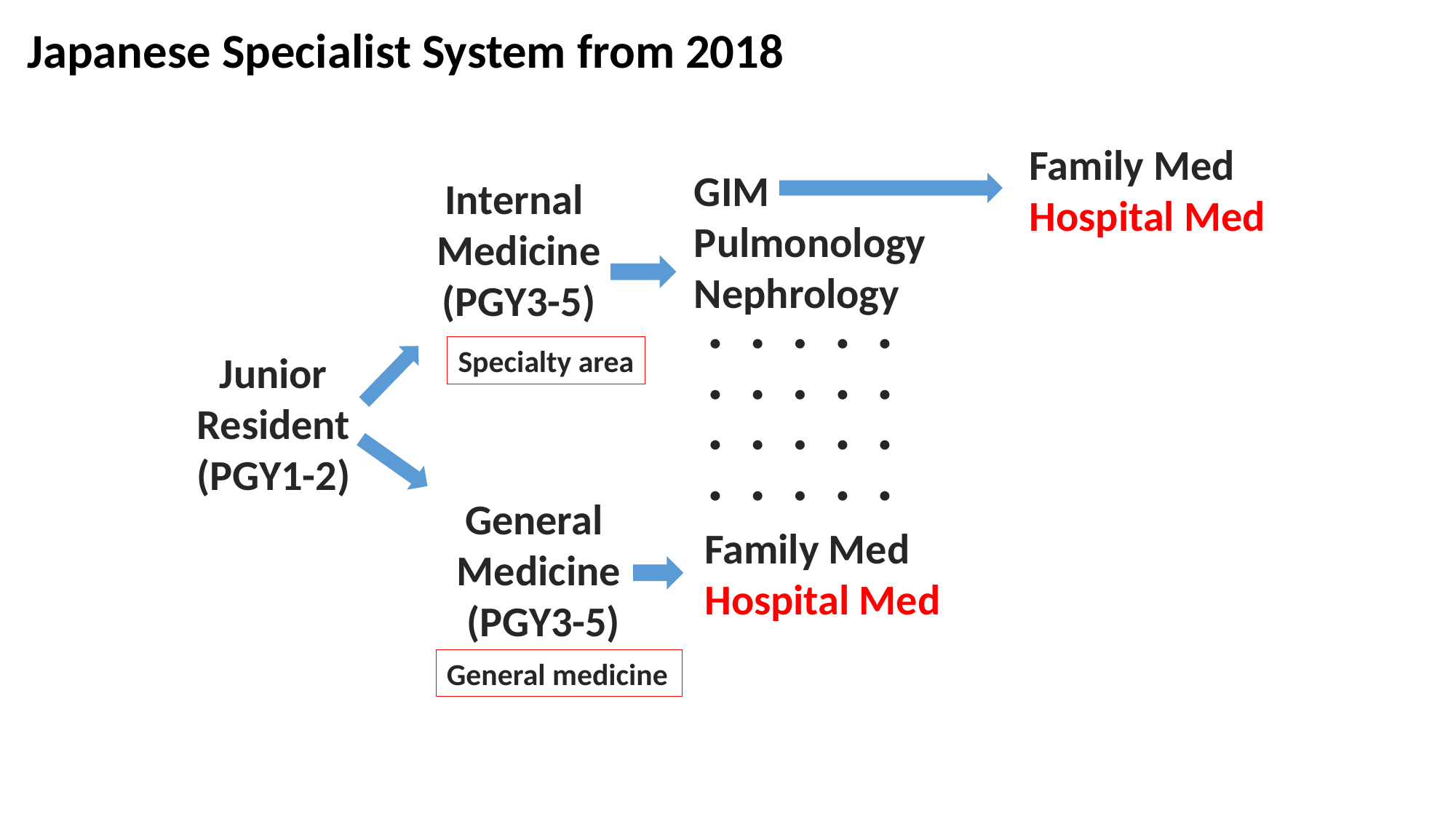

Japanese Specialist System from 2018
Japanese Specialist System from 2018
Family Med
Hospital Med
GIM
Pulmonology
Nephrology
・・・・・・・・・・
・・・・・・・・・・
Internal
Medicine
(PGY3-5)
Specialty area
Junior
Resident
(PGY1-2)
General
Medicine
 (PGY3-5)
Family Med
Hospital Med
General medicine
