# Supplementary material for: Roles considered important for hospitalist and non-hospitalist generalist practice in Japan: a survey study
Source: BMC Prim Care. 2023 Jul 7;24:139. doi: 10.1186/s12875-023-02090-w (PMC10327327; doi:10.1186/s12875-023-02090-w)
Supplement: Supplementary file 2 — Additional file 2: Appendix 2. Questionnaire raw data English Ver. [file 12875_2023_2090_MOESM2_ESM.docx]

**Appendix 2: Questionnaire raw data English Ver.**

**QUESTIONNAIRE**

1. What is your gender?

Man Woman

2. How old are you? _________

3. What year did you graduate from university? __________

4. Are you a member of the Japanese Society of Hospital General Medicine?

Yes No

5. Are you a member of the Japanese Primary Care Association?

Yes No

6. How many beds does your hospital have?

a. 0–19 b. 20–199 c. 200–399 d. 400 and over

7. Which environment do you work in?

a. Clinic b. City hospital c. University hospital

8. What is your position?

a. Resident of specialty training b. Attending physician c. Manager d. Other

8-2. If you have selected other, please indicate your position.

_________________________________________________________

9. Do you consider yourself a general medicine doctor?

Yes No

10. Do you work in a department of general internal medicine or general medicine?

Yes No

11-1. Please choose what is the most important to you as a hospitalist.

a. Inpatient medical management (rehabilitation, nutrition, etc.) b. Palliative care c. Patient education d. Patient Safety(Diagnostic Safety) e. Evidence-based medicine f. Diagnostic Reasoning g. Patient Safety(Medication) h. Education for residents i. Education for staff j. Education for students k. Lifelong self-education l. Inter-professional work m. Communication with medical staff n. Cooperation with other departments o. Hospital management p. Out-of-hospital activities q. Leadership r. Patient Safety(Infection control) s. Research t. Elderly care u. Social work v. Professionalism and medical ethics w. Emergency visit x. First outpatient visit y. Home visit care z. Preventive Medicine (Primary, Secondary and Tertiary)

11-2. Please choose what is the second most important to you as a hospitalist.

a. Inpatient medical management (rehabilitation, nutrition, etc.) b. Palliative care c. Patient education d. Patient Safety(Diagnostic Safety) e. Evidence-based medicine f. Diagnostic Reasoning g. Patient Safety(Medication) h. Education for residents i. Education for staff j. Education for students k. Lifelong self-education l. Inter-professional work m. Communication with medical staff n. Cooperation with other departments o. Hospital management p. Out-of-hospital activities q. Leadership r. Patient Safety(Infection control) s. Research t. Elderly care u. Social work v. Professionalism and medical ethics w. Emergency visit x. First outpatient visit y. Home visit care z. Preventive Medicine (Primary, Secondary and Tertiary) aa. None

11-3. Please choose what is the third most important to you as a hospitalist.

a. Inpatient medical management (rehabilitation, nutrition, etc.) b. Palliative care c. Patient education d. Patient Safety(Diagnostic Safety) e. Evidence-based medicine f. Diagnostic Reasoning g. Patient Safety(Medication) h. Education for residents i. Education for staff j. Education for students k. Lifelong self-education l. Inter-professional work m. Communication with medical staff n. Cooperation with other departments o. Hospital management p. Out-of-hospital activities q. Leadership r. Patient Safety(Infection control) s. Research t. Elderly care u. Social work v. Professionalism and medical ethics w. Emergency visit x. First outpatient visit y. Home visit care z. Preventive Medicine (Primary, Secondary and Tertiary) aa. None
